# Enhancing heat transfer in solar-powered ships: a study on hybrid nanofluids with carbon nanotubes and their application in parabolic trough solar collectors with electromagnetic controls

**DOI:** 10.1038/s41598-023-36716-x

**Published:** 2023-06-10

**Authors:** A. M. Obalalu, M. Asif Memon, O. A. Olayemi, J. Olilima, Amsalu Fenta

**Affiliations:** 1 Department of Mathematical Sciences, Augustine University Ilara-Epe, Lagos, Nigeria; 2 Department of Mathematics and Social Sciences, Sukkur IBA University, Sukkur, 65200 Sindh Pakistan; 3 School of Engineering, Cranfield University, Cranfield, UK; 4 Department of Aeronautics and Astronautics, Kwara State University, Malete, 23431 Nigeria; 5 Department of Physics, Mizan Tepi University, PO Box 121, Tepi, Ethiopia

**Keywords:** Engineering, Materials science, Mathematics and computing

## Abstract

The aim of this research is to explore the use of solar-powered ships (SPS) as a means to reduce greenhouse gas emissions and fossil fuel dependency in the maritime industry. The study focuses on improving the heat transfer efficiency in SPS by employing hybrid nanofluids (HNF) containing carbon nanotubes (CNTs). Additionally, a novel approach utilizing renewable energy and electromagnetic control is proposed to enhance the performance of SPS. The research implements the non-Newtonian Maxwell type and Cattaneo–Christov heat flux model in parabolic trough solar collectors used for ships. The study conducts theoretical experiments and simulations to evaluate the thermal conductivity and viscosity of the CNT-based HNF. Various properties, including solar thermal radiation, viscous dissipation, slippery velocity, and porous media, are assessed to determine the effectiveness of thermal transport in SPS. The research employs similarity variables to simplify the complex partial differential equations into ordinary differential equations and solves them using the Chebyshev collocation spectral method. The results indicate that the MWCNT-SWCNT/EO hybrid nanofluid significantly improves the thermal conductivity, thereby enhancing heat transfer. The HNF exhibits an efficiency rate of approximately 1.78% with a minimum efficiency rate of 2.26%.

## Introduction

The global energy demand continues to rise, but the depletion of fossil fuels and the increasing cost of traditional energy sources, such as electricity, have led scientists to shift their focus toward renewable energy in recent years^1^. Generating electricity from sustainable sources is an eco-friendly way of producing power since it doesn't release any greenhouse gases. In contrast, the burning of fossil fuels emits carbon dioxide into the atmosphere, contributing to global warming^2^. In addition, environmentalists strongly believe that using sustainable resources can have a significant impact on decreasing carbon pollution and slowing down the rate of global warming^3^. In recent years, solar energy has become a widely-discussed option for sustainable energy due to its accessibility, lack of harmful emissions, and minimal environmental impact, making it a highly attractive choice for energy production^4^. Solar energy has the highest potential for long-term use, accessibility, and the least negative impact on the environment among all energy sources. With proper utilization, this source of energy has the potential to generate roughly four times the amount of power that is currently being used worldwide^5^. Recent studies have shown that global CO2 emissions are expected to decrease by 75% by 2050, compared to the levels recorded in 1985^6^. According to a study carried out by the US Department of Energy, the amount of solar energy that reaches the earth's surface in just 90 min is sufficient to meet the world's energy demands for an entire year^7^. In a report from the United States, it was discovered that solar energy can serve as a natural substitute for other forms of energy. Additionally, solar power systems can generate thermal energy that can be utilized for heating and cooling purposes. Therefore, the main concern now is figuring out the most effective way to harness the sun's energy.

Two prominent methods for converting sunlight into electricity are currently widely used and should be familiar to you: photovoltaic systems (PV) and concentrated solar energy (CSE)^8^. PV systems have numerous applications, including (a) Generating electricity for homes and businesses^9^ (b) Providing power for spacecraft and satellites^10^ (c) Powering various types of vehicles such as cars, buses, and boats, and (d) Remote Area Power Supply (RAPS) system which consists of generators, streetlights, and wireless communication devices that provide electricity to areas located far away from urban centers. In brief, PV systems provide an eco-friendly, affordable, and low-maintenance way to produce electricity that is sustainable and can work independently of the power grid. A photovoltaic device primarily consists of a semiconductor material that conducts electricity through electrons. Unlike conductors, which can conduct an unlimited number of electrons, semiconductors possess unique properties that enable them to regulate the flow of electrons. This feature makes semiconductors valuable components for photovoltaic devices. Meanwhile, CSP is a method of generating electricity from the sun's rays by using reflective surfaces or optical technology to concentrate and focus sunlight onto a very small area. This concentrated sunlight is converted into heat, which can be used to create steam that powers a turbine and generates electricity^11^. CSP plants can produce large amounts of electricity, and they are particularly beneficial in areas with high levels of solar radiation. Additionally, these plants can be built on a large scale. To generate electricity from solar energy, concentrated solar power technology is used which involves focusing the sun's rays onto a small area through reflectors or optics. This process creates heat that can then be used to produce electricity by passing it through an electrical generator. However, it is important to note that not all technologies rely on mirrors to concentrate sunlight, as some use optics or other systems. It should also be noted that the conversion of sunlight to heat occurs through a physical process called absorption^12^. According to ref^13^, areas characterized by dry and hot climates such as California and Arizona in the United States are conducive to the development of larger plants that utilize concentrated solar power. This form of renewable energy is preferred over non-renewable alternatives such as fossil fuels due to its eco-friendliness and lack of emissions of harmful pollutants. In conclusion, solar energy is a clean and sustainable option that is preferred over traditional energy sources.

**Figure 1:**
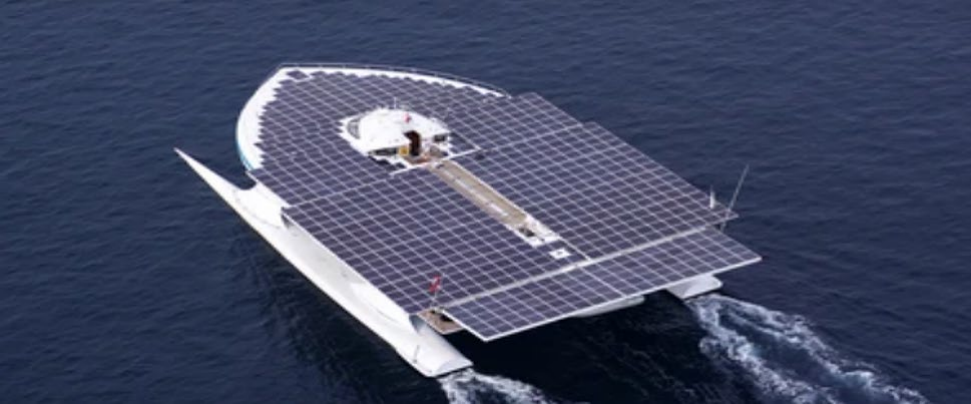
Depicts the solar-powered ship.

Currently, the utilization of heavy fuel oil (HFO) and marine diesel oil (MDO) for industrial transportation is responsible for roughly 3% of the total global carbon dioxide emissions^14^. Based on reference^15^, it was reported that approximately 932 million metric tons of pollutants were discharged into the air during the year 2015. To put this into perspective, this amount is more than the total pollution generated by Germany in the year 2017, which was approximately 905 million tonnes. The pollutants released by these ships contain numerous harmful contaminants that can negatively impact human health, the environment, and the climate. Furthermore, reference^16^ emphasizes that the discharge of gases from the combustion of heavy fuel oil includes a mixture of sulfur oxide, combustion byproducts, nitrogen oxides, and heavy metals, as well as carbon dioxide. In recent times, researchers have been investigating the possibility of using solar energy to power commercial ships. The goal is to reduce the number of greenhouse gases produced by these vessels. The use of solar energy in powering ships is seen as a desirable option by many countries because it allows for the creation of eco-friendly vessels. In the nineteenth century, French scientist Augustin Mouchot was the first person to propose the idea of utilizing solar energy as a source of propulsion for ships^17^. The first operational solar-powered vessels were constructed in the 1970s, and their main objective was for recreational activities. In 1990, Switzerland was the location where the Sun 21 was assembled, which was the first commercial transport powered by solar energy. This boat was a catamaran that had two electric engines, which were powered by solar panels mounted on the surface of the boat. Following this groundbreaking development, numerous solar-powered ships have been developed and utilized in various industries, such as cargo transport, as well as scientific research^18^. The main objective behind the development of solar-powered ships is to reduce reliance on fossil fuels and mitigate the environmental impact of shipping. The shipping industry is responsible for a significant amount of greenhouse gas emissions, and solar-powered ships offer a cleaner and more sustainable alternative. By harnessing the power of the sun, these ships can reduce their carbon footprint and provide a more environmentally friendly mode of transportation. According to reference^19^, the International Maritime Organization (IMO) has set a target to reduce greenhouse gas emissions from transportation by at least 50% by the year 2050. This target is part of the IMO’s efforts to address climate change and promote sustainable development in the maritime industry. The study conducted by Obalalu et al.^20^ focuses on evaluating the efficiency of thermal transfer in a solar-powered ship that uses mono/hybrid nanofluids for solar water pumps. The analysis takes into account solar radiation as the primary source of heat and examines different factors such as thermal radiative flowing, viscous dissipation, and heat source to determine the ship's performance. It was concluded that the relative proportion of heat transmission rate is increased by 24% in hybrid nanofluid when compared to nanofluid. The research work of Bellos et al.^21^ emphasizes the significance of carrying out comprehensive thermal and entropy analysis in solar-thermal systems. Furthermore, the study suggests the utilization of nanofluids to upgrade the performance of parabolic trough solar collectors (PTSCs). The study concluded that there is a significant increase in the relative heat transmission rate when the permeability of the permeable medium increases from 1.6 to 14.9%. According to Kirkpatrick’s^22^ study on Navy surface combatant ships, the efficiency of the photovoltaic systems installed was evaluated. The study findings revealed that the additional weight caused by the installation of solar cells is insignificant compared to the weight of critical supplies, including fuel and food, that are indispensable for ships during voyages. A study conducted by^23^ investigated the feasibility of using solar panels as a source of power on small fishing vessels that operate in remote areas. The results indicate that the placement of solar panels on the boat may cause extra drag, which could increase fuel consumption, particularly in windy conditions. Hussein et al.^24^ conducted research that indicates the feasibility of utilizing photovoltaic systems as a means of generating renewable energy on ships, thereby reducing the reliance on non-renewable fossil fuels. In the study of Lan et al.^25^, the researchers introduced a methodology that aims to identify the optimal proportion of photovoltaic system integration for ship power operations. The goal of this approach is to reduce financing expenses, fuel costs, and engine emissions. Additionally, they conducted further investigations to improve the slope-angle characteristic of the photovoltaic panels installed on a large oil carrier vessel. Figure 1 illustrates a ship that is powered by solar energy.

In recent years, nanofluids have become increasingly popular as a working fluid for various heating applications, including solar collectors. This is due to their effectiveness in enhancing heat transfer^26^. Parabolic trough solar collect (PTSC) is a type of solar energy device that harnesses the sun's energy by using a concave mirror to reflect and concentrate sunlight onto a tube containing a liquid, which in turn absorbs the heat and converts it into usable energy. It is common practice to use a heat transfer fluid (HTF) to fill the receiver tube^27^. The purpose of the HTF is to transfer thermal energy to a heat exchanger, where it is employed to create steam to produce electricity. Multiple research studies have found that using nanofluids as heat transfer fluids in parabolic trough solar collectors can boost the overall effectiveness of the system. This is due to the superior thermal properties of nanofluids compared to traditional fluids. These properties include higher thermal conductivity and specific heat capacity. Additionally, the presence of nanoparticles in nanofluids leads to an increased surface area for heat transfer, ultimately improving the system’s effectiveness^28^. The improved performance of parabolic trough solar collectors achieved through the utilization of nanofluids has significant repercussions for the development of sustainable energy sources in the future. By using more efficient working fluids, the energy produced by sunlight can be increased. This approach can lead to a decrease in reliance on natural fuels, which in turn can aid in mitigating the impacts of climate change.

Nanofluids (NF) are known for their distinctive thermal properties, which can significantly improve the heat transfer efficiency of solar power systems (SPS)^29^. Nanofluid (NF) is a term used to describe a fluid mixture that contains nanoparticles (NPs) of metallic (Gold, Titanium dioxide, Iron) or non-metallic (polyethylene glycol, zinc oxide, Silica) materials suspended in a base-fluid (BF). Nanofluids are a promising option for thermal applications due to various key factors. These factors include the ability to conduct heat efficiently, increase the surface area, improve specific heat capacity, ensure stability, and reduce viscosity^30^. The combination of these characteristics makes nanofluids a compelling choice for use in many industries. Carbon nanotubes (CNTs), which are a type of nanoparticle made mostly of carbon atoms. CNTs have a cylindrical shape and come in various lengths, ranging from a few nanometers to several micrometers^31^. The unique physical, mechanical, and electrical properties of CNTs make them suitable for use in diverse fields, including medicine, electronics, and energy storage. (CNTs) are classified into various types, such as single-walled carbon nanotubes (SWCNTs), double-walled carbon nanotubes (DWCNTs), and multi-walled carbon nanotubes (MWCNTs)^32^. SWCNTs are composed of a solitary cylindrical structure comprised of carbon atoms. Conversely, MWCNTs and DWCNTs are composed of multiple nested cylinders of carbon atoms. The structural and compositional characteristics of individual types of carbon nanotubes (CNTs) confer distinct properties which make them useful across a wide range of fields^33^. Salawu et al.^34^ conducted a study to examine the elastic deformation of a mixture of magneto-nanofluids that contain SWCNTs coated with silver nanoparticles and MWCNTs functionalized with molybdenum disulfide nanoparticles within a porous cylinder. Hachicha et al.^35^ conducted a study to investigate the effectiveness of using a type of nanofluid made of MWCNT and water to enhance the heat transfer in PTSC under different seasonal conditions. Their computer simulations showed that the use of this nanofluid led to a significant increase of up to 21 percentage points in the Nusselt number, which is a key parameter used to measure the rate of heat transfer. The recent work of^36^ studies the electromagnetic couple stress of single and multiwall carbon nanotubes (SWCNTs, MWCNTs) upon a rotary disc under the influence of chemical reactions. Heat transfer mechanism in ternary nanofluid between parallel plates channel using modified Hamilton-Crossers model and thermal radiation effects was study by^37^. Numerical study of thermal enhancement in ZnO-SAE50 nanolubricant over a spherical magnetized surface influenced by Newtonian heating and thermal radiation was study by^38^. Analysis of heat transfer performance for ternary nanofluid flow in radiated channel under different physical parameters using GFEM was study by^39^. Heat transfer inspection in [(ZnO-MWCNTs)/water-EG(50:50)]hnf with thermal radiation ray and convective condition over a Riga surface were study by^40^. Thermal efficiency in hybrid (Al2O3–CuO/H2O) and ternary hybrid nanofluids (Al2O3–CuO–Cu/H2O) by considering the novel effects of imposed magnetic field and convective heat condition was study by^41^. Numerical thermal featuring in gAl2O3–C2H6O2 nanofluid under the influence of thermal radiation and convective heat condition by inducing novel effects of effective Prandtl number model (EPNM) was study by^42^.

A hybrid nanofluid (HNF) is a composite fluid consisting of a base fluid and nanoparticles, which is utilized to enhance the heat transfer characteristics of the fluid^43^. HNF has been identified as a potential means of improving the efficacy of solar collectors and solar cells with radiation from the sun. Solar collectors are devices that gather energy from the sun, transform it into heat, and then transfer it to a liquid that flows within the collector. The hybrid nanofluids contain tiny particles that can enhance the heat transfer efficiency of the fluid by increasing its thermal conductivity. This improvement has the potential to enhance the overall performance of the solar collector and convert solar radiation into useful energy at a faster rate^36^. Solar cells generate a lot of heat when they convert solar radiation into electrical energy. To tackle this issue, hybrid nanofluids can be used as a cooling agent. By mixing nanofluids, this can be easily accomplished.

Using HNF in solar energy systems can prevent photovoltaic cells from overheating, which improves their efficiency and lifespan. Incorporating hybrid nanofluids in solar energy systems can increase the efficiency of heat transfer and enhance the rate at which solar radiation is converted into useful energy. Although there are challenges to using hybrid nanofluids in solar energy systems, researchers are currently working to overcome them. The goal is to improve the efficiency and safety of these nanofluids. Even though there are obstacles, hybrid nanofluids have the potential to make solar energy systems more efficient and better for the environment. In regards to this, the work of Khan et al.^44^ emphasized the thermal Performances of radiative viscous copper-alumina with ethylene glycol/water HNF across porous stretched cylinders. The mathematical modeling of MHD copper oxide–iron oxide/blood HNF vertical porous channel under the influence of thermal radiation was investigated by^45^. Their result shows that blood flow is greater for copper oxide NF than the copper oxide–iron oxide HNF. Numerical assessment of a sutterby hybrid nanofluid over a stretching sheet with a particle shape factor was investigated by ^46^. Entropy generation and thermal performance of Williamson hybrid nanofluid flow used in solar aircraft application as the main coolant in parabolic trough solar collector was investigated by^47^. Solar-HVAC thermal investigation utilizing (Cu-AA7075/C6H9NaO7) MHD-driven hybrid nanofluid rotating flow via second-order convergent technique: A novel engineering study was investigated by^48^. Galerkin finite element solution for electromagnetic radiative impact on viscid Williamson two-phase nanofluid flow via extendable surface was investigated by^49^. Irreversibility analysis of time-dependent magnetically driven flow of Sutterby hybrid nanofluid: a thermal mathematical model was investigated by^50^. Dynamics of radiative Williamson hybrid nanofluid with entropy generation: significance in solar aircraft was investigated by^51^. Dynamics of heat absorbing and radiative hydromagnetic nanofluids through a stretching surface with chemical reaction and viscous dissipation was investigated by^52^. Thermal-enhanced hybrid of copper–zirconium dioxide/ethylene glycol nanofluid flowing in the solar collector of water-pump application was investigated by^53^. Chemical reaction and thermal characteristiecs of Maxwell nanofluid flow-through solar collector as a potential solar energy cooling application: A modified Buongiorno's model was investigated by^54^. Dynamics of ethylene glycol-based graphene and molybdenum disulfide hybrid nanofluid over a stretchable surface with slip conditions was investigated by^55^. Numerical and statistical explorations on the dynamics of water conveying Cu-Al2O3 hybrid nanofluid flow over an exponentially stretchable sheet with Navier’s partial slip and thermal jump conditions was investigated by^56^. Mechanical improvement in solar aircraft by using tangent hyperbolic single-phase nanofluid was investigated by ^57^. Implication of Arrhenius activation energy and temperature-dependent viscosity on non-Newtonian nanomaterial bio-convective flow with partial slip was investigated by^58^. Thermal analysis characterisation of solar-powered ship using Oldroyd hybrid nanofluids in parabolic trough solar collector: An optimal thermal application was investigated by^59^.

Viscosity is a fundamental concept in fluid mechanics that refers to a substance's resistance to movement. This principal divides fluids into two categories: Newtonian and non-Newtonian fluids (NNF). Newtonian fluids have a fixed viscosity that doesn't change with the amount of force or pressure applied to them. Examples of Newtonian fluids include water, air, and various oils^60, 61^. On the other hand, non-Newtonian fluids have a different kind of behavior where their viscosity changes depending on the conditions of the flow and the amount of pressure or force exerted on them^62^. So, the viscosity of non-Newtonian fluids varies based on the flow conditions they are subjected to. The NNF is present in a diverse range of natural and artificial systems including dietary products, cosmetics, pharmaceuticals, and geological and biological systems. Some common examples of NNF are ketchup, toothpaste, and blood. One of the most significant variables that can affect the thickness of NNF is the amount of solar radiation that the substance is exposed to. Solar radiation, particularly in the form of ultraviolet (UV) rays, can cause changes in both the molecular structure and physical properties of NNF^63^. These changes may affect the viscosity and flowability of the material. Research findings suggest that non-natural fibers, including polymers and proteins, may undergo modifications in their molecular structure when exposed to UV radiation. The properties of NNF, such as their ability to flow and elasticity, can be influenced by light energy, which can change their temperature. When NNF is exposed to UV radiation, it can break down, reducing its thickness and effectiveness. In geological systems, sunlight can heat NNF, making it less viscous and easier to move^64^. The Maxwell fluid is a particular kind of NNF that exhibits both viscous and elastic properties when subjected to stress, making it unique among other non-Newtonian fluids^65^. Maxwell fluids are frequently used in the modeling of viscoelastic materials such as polymer solutions, jellies, and colloidal suspensions. The behavior of these fluids was characterized by the Scottish scientist James Clerk Maxwell^66^. The effectiveness of a hybrid nanoparticle made of a viscoelastic coolant Maxwell fluid and carbon nanotubes in PTCS was studied by^67^. The thermal characteristics of a two-dimensional flow of Maxwell nanofluid over a permeable stretching sheet were analyzed by the researchers^68, 69^ using the finite element method. The objective of this study was to improve understanding of the behavior of these substances and their potential applications.

The Cattaneo–Christov heat flux is a modified form of Fourier's law of heat conduction, which considers the fact that materials take some time to attain thermal equilibrium. In 1948, Cattaneo developed a modified equation for heat transmission that considered a relaxation period^70^. In 1977, Christov further enhanced this equation by introducing an additional component that accounted for the effects of viscosity. The use of Cattaneo–Christov heat flux in the study of solar energy is a recent development that started in the 1990s. Since then, integrating this heat flux into solar models has led to significant advancements in understanding the Sun's internal structure and dynamics. In the field of solar physics, the Cattaneo–Christov heat flux has played a vital role in resolving long-standing issues. By including this in solar models, scientists have observed an improvement in the accuracy of their empirical observations. This has led to a better understanding of the internal structure and dynamic characteristics of the Sun, resulting in increased precision in solar modeling^71^. The studies mentioned in Reference^72, 73^ provide further information on the Cattaneo–Christov heat flux.

**Figure 2:**
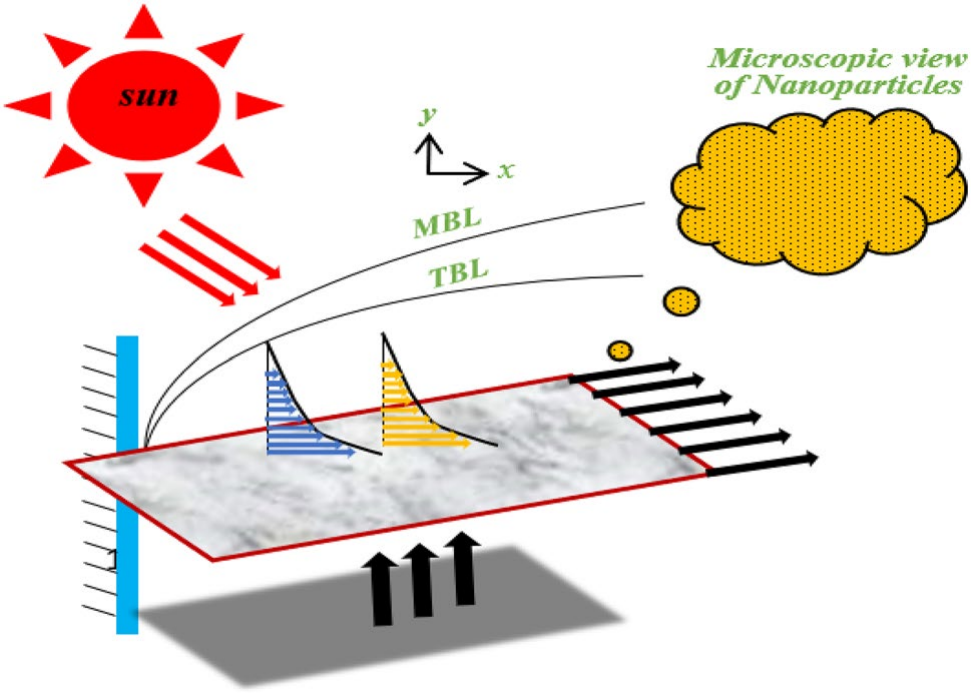
Geometrical diagram of the flow.

The use of solar energy is important for developing nations and eco-friendly sources of power. Solar-powered ships and boats have numerous advantages such as affordability, noise reduction, continuous charging, and the ability to charge personal devices. Additionally, they have minimal environmental impacts and are highly dependable. Research conducted in various studies has highlighted the significance of conducting a thorough analysis of non-Newtonian and thermal factors in solar-thermal systems. Moreover, incorporating hybrid nanofluids in PTSCs is crucial to enhance the operational efficiency of the system. The originality of the present research lies in investigating the flow characteristics of a viscous Maxwell hybrid nanofluid over a horizontal surface subjected to infinite heat flux, employing the Cattaneo–Christov model for analysis. The primary objective is to enhance the thermal efficiency of PTSCs. To achieve this, two types of Maxwell nanofluids are utilized: Single-walled carbon nanotubes-Maxwell nanofluids and multi-walled carbon nanotubes/Engine oil (MWCNT-SWCNT/EO). The distinctiveness of this study lies in its focus on solar-powered ships and boats, examining the impact of dimensionless numbers on entropy production. By conducting a comprehensive analysis of non-Newtonian and thermal factors in solar-thermal systems and investigating the specific application of hybrid nanofluids in PTSCs, this research contributes to the understanding and advancement of PTSCs’ operational efficiency in the context of solar-powered maritime transportation. Figure 2 provides a graphical representation of the PTSC.

The primary aim of creating Fig. 2 is to demonstrate the sequential process of developing the current theoretical model. The diagram outlines how solar energy reaches the PTSC and travels through a fluid accompanied by thermal radiation, resulting in the maximum energy storage on the PTSC through thermal conductivity. The stored energy is in the form of heat, which is then transformed into electrical energy by photovoltaic cells in a battery. This electrical energy is used for various purposes within the solar-powered ship.

1. The PTSC’s cylindrical surface receives solar energy from the sun, which is then transformed into heat energy. The presence of nanoparticles in the fluid that flows through the PTSC intensifies this heat energy. The central aim of the current theoretical experiment is to enhance the PTSC’s heat storage capacity by incorporating physical phenomena such as thermal radiation and conductivity.
2. The surface of PTSC receives a high amount of solar energy in the form of heat, which can be converted into electrical energy to power navigation and lighting systems. This is achieved using a solar cell battery located in the spacecraft's fuel area box, which can transform the heat energy into electrical energy. During the day, the battery stores the energy generated, which is then used to power the spacecraft at night.
3. The energy stored in a battery is a critical factor in determining its ability to power various functions such as avionics, navigation lamps, and military communication. The battery’s energy storage capacity directly affects its ability to operate these functions effectively. Therefore, it is important to consider the battery’s energy storage capacity when choosing a battery for specific applications.

## Flow model of the problem

### Physical description

**Figure 3:**
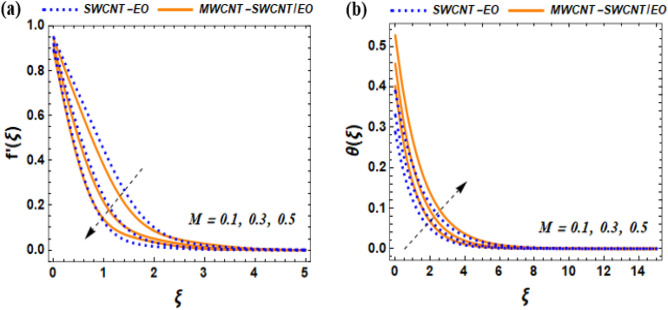
Effect of $M$ on ${f}^{^{\prime}}\left(\xi \right)$, $\theta \left(\xi \right)$.

The mathematical model describing fluid flow in this system demonstrate the non-uniform stretching velocity on a solid surface, such as a flat plate moving horizontally through a fluid. An equation that represents this is ${U}_{w}$(x, 0) = $bx$, where the parameter $b$ denotes the initial ratio of expansion. The temperature of an insulated surface is denoted by ${\Theta }_{w}$($x$, t) = ${\Theta }_{\infty }+{b}^{*}x$ and is assumed to remain constant at $x$ = 0. The rate of thermal change is denoted as ${b}^{*}x$, while ${\Theta }_{w}$ refers to the temperature of the wall and ${\Theta }_{\infty }$ represents the surrounding temperature. The plate is having a slippery surface that experiences a temperature change. Figure 3 presents a schematic representation of the flow.

### Suppositions and terms of the system

The mathematical formulation has been developed based on the assumptions presented below:

- Non-Newtonian Maxwell nanofluid, Cattaneo–Christov heat flux.
- Multi-walled and single-walled carbon nanotubes (MWCNT and SWCNT).
- Flow has viscid dissipation properties, a Porous medium, and Heat generation.
- Slippery boundary constraints, Entropy generation, Steady flow conditions.
- Solar thermal radiation, Magnetic field.
- Boundary-layer approximations, Engine oil (EG) as BF.

### Governing equations

Given the assumptions stated earlier, the equations that govern the non-Newtonian Maxwell model with radiation heat flux can be expressed as follows^12^:

1 $$\frac{\partial {A}_{1}}{\partial x}+\frac{\partial {A}_{2}}{\partial y}=0$$

2 $${A}_{1}\frac{\partial {A}_{1}}{\partial x}+{A}_{2}\frac{\partial {A}_{1}}{\partial y}= \frac{{\mu }_{hnf}}{{\rho }_{hnf}}\left(\frac{{\partial }^{2}{A}_{1}}{\partial {y}^{2}}\right)-\varsigma \left({{A}_{1}}^{2}\frac{{\partial }^{2}{A}_{1}}{\partial {x}^{2}} +{{A}_{2}}^{2}\frac{{\partial }^{2}{A}_{2}}{\partial {y}^{2}}+ 2{A}_{1}{A}_{2}\frac{{\partial }^{2}{A}_{2}}{\partial x\partial y}\right)-\frac{{\sigma }_{hnf}{B}_{0}^{2}}{{\rho }_{hnf}}{A}_{1}-\frac{{\mu }_{hnf}}{{\rho }_{hnf}k}{A}_{2}$$

3 $$\begin{aligned} A_{1} \frac{{\partial {\Theta }}}{\partial x} + A_{1} \frac{{\partial {\Theta }}}{\partial y} & = \frac{{k_{hnf} }}{{\left( {\rho C_{p} } \right)_{hnf} }}\left( {\frac{{\partial^{2} {\Theta }}}{{\partial y^{2} }}} \right) + \frac{{\mu_{hnf} }}{{\left( {\rho C_{p} } \right)_{hnf} }}\left( {\frac{{\partial \nu_{1} }}{\partial x}} \right)^{2} + \frac{1}{{\left( {\rho C_{p} } \right)_{hnf} }}Q\left( {{\Theta } - {\Theta }_{w} } \right) \\ & \quad + \omega \left( {A_{1} \left( {\frac{{\partial A_{2} }}{\partial x}\frac{{\partial {\Theta }}}{\partial x}} \right) + A_{1} \left( {\frac{{\partial A_{1} }}{\partial y}\frac{{\partial A_{2} }}{\partial y}} \right) + A_{1} \left( {\frac{{\partial \nu_{2} }}{\partial x}\frac{{\partial {\Theta }}}{\partial y}} \right) + A_{1} \left( {\frac{{\partial A_{1} }}{\partial y}\frac{{\partial {\Theta }}}{\partial x}} \right)} \right. \\ & \quad \left. { + A_{1}^{2} \left( {\frac{{\partial^{2} {\Theta }}}{{\partial x^{2} }}} \right) + A_{2}^{2} \left( {\frac{{\partial^{2} {\Theta }}}{{\partial y^{2} }}} \right) + 2A_{1} A_{2} \frac{{\partial^{2} {\Theta }}}{\partial x\partial y}} \right) \\ & \quad - \frac{1}{{\left( {\rho C_{p} } \right)_{hnf} }}\left( {\frac{{\partial q_{r} }}{\partial y}} \right) + \frac{{\sigma_{hnf} B_{0}^{2} }}{{\left( {\rho C_{p} } \right)_{hnf} }}A_{1}^{2} \\ \end{aligned}$$

with boundary conditions^74^:

4 $$\begin{aligned} A_{1} ({\text{x}},0) & = U_{w} + P_{w} \left( {\frac{{\partial A_{1} }}{\partial y}} \right),\quad A_{2} ({\text{x}},0) = R_{{\text{w}}} , \\ & \quad - k_{g} \left( {\frac{{\partial {\Theta }}}{\partial y}} \right) = h_{g} \left( {{\Theta }_{w} - {\Theta }} \right),\quad A_{1} \to 0, \\ & \quad {\Theta } \to {\Theta }_{\infty } ,\;{\text{as}}\quad y \to \infty . \\ \end{aligned}$$

Table 1 provides information on the thermophysical characteristics of the nanofluid as well as the symbols utilized in the current research. When a surface is heated through convection, it is important to take into account the amount of heat that is lost through conduction, also known as Newtonian heating. It is also important to understand the behavior of the fluid close to the surface, which can be affected by the slip condition. This refers to how fluids behave when they come into contact with solid boundaries and can affect the rate at which the fluid moves near the surface. The extent of the slip is directly related to the amount of shear stress experienced by the fluid at the boundary^75, 76^. This understanding is crucial for accurately analyzing heat transfer through convection.

**Table 1:** The thermophysical factors of the SWCNT, MWCNT nanoparticles, and engine oil base fluid^78^.

Thermophysical	$C_{p}$/(${\text{J}}\;{\text{kgK}}$)	$k$/(${\text{W}}\;{\text{mK}}$)	$\rho$/(${\text{kg}}\;{\text{m}}^{ - 3}$)	$\sigma$(S ${\text{m}}^{ - 1}$)
MWCNT	796	3000	1600	10^6^–10^−7^
SWCNT	425	6000	2600	10^−6^–10^10^
Engine oil (EO)	1910	0.14	884	10^−11^–10^−9^

### Thermodynamics physical variables of nanofluid (NF) and hybrid nanofluid (HNF)^77^

HNF consists of a base fluid and two-element nanoparticles. They are utilized to enhance the heat transfer ability of regular fluids and possess a greater heat exponent compared to NF. The Maxwell HNF thermodynamics physical variables are listed below.

*NF:*

$${\left(\rho {C}_{p}\right)}_{nf}=\left(1-\phi \right){\left(\rho {C}_{p}\right)}_{f}-\phi {\left(\rho {C}_{p}\right)}_{s}$$

$$\frac{{k}_{hnf}}{{k}_{gf}}=\frac{({k}_{s}+2{k}_{f})-2\varphi \left({k}_{f}-{k}_{\mathrm{s}}\right)}{({k}_{s}+2{k}_{f})+\varphi \left({k}_{f}-{k}_{\mathrm{s}}\right)}$$

$${\sigma }_{nf}={\sigma }_{f}\left(1+\frac{3\left(\frac{{\sigma }_{s}}{{\sigma }_{f}}-1\right)\phi }{2+\frac{{\sigma }_{s}}{{\sigma }_{f}}-\left(\frac{{\sigma }_{s}}{{\sigma }_{f}}-1\right)\phi }\right)$$

$${\mu }_{nf}=\frac{{\mu }_{f}}{{\left(1-\phi \right)}^{2.5}}$$

$${\rho }_{nf}=\left(1-\phi \right){\rho }_{f}-\phi {\rho }_{s}$$

*HNF*:

$$\rho_{hnf} = \left( {1 - (\phi_{1} + \phi_{2} )} \right)\rho_{f} + \phi_{1} \rho_{{s_{1} }} + \phi_{2} \rho_{{s_{2} }}$$

$$\frac{{k}_{hnf}}{{k}_{nf}}=\left(\frac{({k}_{\mathrm{s}2}+2{k}_{nf}-{2\phi }_{2}\left({k}_{nf}-{k}_{\mathrm{s}2}\right)}{({k}_{\mathrm{s}2}+2{k}_{nf}+{\phi }_{2}\left({k}_{nf}-{k}_{\mathrm{s}2}\right)}\right)$$

$$\frac{{k}_{nf}}{{k}_{f}}=\left(\frac{{k}_{\mathrm{s}1}+2{k}_{f}-2{\phi }_{1}\left({k}_{f}-{k}_{\mathrm{s}1}\right)}{({k}_{\mathrm{s}1}+2{k}_{f}+{\phi }_{1}\left({k}_{f}-{k}_{\mathrm{s}1}\right)}\right)$$

$$\frac{{\sigma }_{hnf}}{{\sigma }_{f}}=1+\frac{3\left(\frac{{\phi }_{1}{\sigma }_{1}+{\phi }_{2}{\sigma }_{2}}{{\sigma }_{bf}}-\left({\phi }_{1}+{\phi }_{2}\right)\right)}{\left(\frac{{\phi }_{1}{\sigma }_{1}+{\phi }_{2}{\sigma }_{2}}{\left({\phi }_{1}+{\phi }_{2}\right){\sigma }_{bf}}+2\right)-\left(\frac{{\phi }_{1}{\sigma }_{1}+{\phi }_{2}{\sigma }_{2}}{{\sigma }_{bf}}-\left({\phi }_{1}+{\phi }_{2}\right)\right)}$$

$${\mu }_{hnf}={{\mu }_{f}/\left(1-{(\phi }_{1}{+\phi }_{2})\right)}^{2.5}$$

$$\left( {\rho C_{p} } \right)_{hnf} = \left( {1 - (\phi_{1} + \phi_{2} )} \right)\left( {\rho C_{p} } \right)_{f} + \phi_{1} \left( {\rho C_{p} } \right)_{{{\text{s}}1}} + \phi_{2} \left( {\rho C_{p} } \right)_{s2}$$

Table 1 displays the thermophysical factors of SWCNT, MWCNT nanoparticles, and engine oil base fluid. Recently, there has been researched exploring how nanomaterials could improve heat exchanger performance^79^. Among various types of nanomaterials, scientists have identified SWCNT and MWCNT as promising for enhancing heat transfer efficiency in heat exchangers. By adding these nanomaterials to the base fluid of engine oil, the heat transfer rate and thermal conductivity of the fluid could be substantially improved, leading to increased heat exchanger efficiency. This method could potentially lead to the creation of more efficient, lightweight, and compact heat exchangers, which would be beneficial for various engineering applications requiring the use of engine oil, including solar-powered ships.

### Rosseland approximation (RA)

The RA is a tool in mathematics utilized to forecast the way energy is transmitted from a surface to the fluid that surrounds it. The Rosseland-approximation method is deemed suitable for cases where there are minor temperature differences between the surface and the fluid. This implies that when the temperature difference is insignificant, the Rosseland-approximation can precisely predict the amount of energy that will be exchanged between the surface and the fluid. Precise forecasts of energy transfer hold great significance in various scientific and engineering domains such as weather forecasting, space science, and materials study^80^. This makes it vital to understand its importance. In addition, the formula for energy is highly non-linear and computationally difficult to explain to temperature ($\Theta$). However, if the temperature differences inside the stream are minimal, a significant simplification can be achieved. This means that the calculation process can be made easier if there are only small thermal variations within the stream. Under certain circumstances, the Rosseland Approximation formula can be simplified by substituting the variable ${\Theta }_{\infty }$ with ${\Theta }^{3}$ to obtain a linear equation to temperature $({{\Theta }_{\infty })}^{3}$. To account for the impact of radiation, the formula provided by Rosseland is used in formula (3), which is represented by the following expression^81^:

5 $$qr = - \frac{{4\sigma^{*} }}{{ 3k^{*} }}\frac{{\partial {\Theta }}}{\partial y},$$

The absorption coefficient, which is represented by k*, and the Stefan Boltzmann constant, symbolized by σ*.

### The problem solves

The equations used for solving boundary value problems (BVP) known as Eqs. (1)–(3) are transformed into simpler, non-dimensional forms using similarity conversions. This conversion technique transforms the PDEs into ODEs. This leads to the expression of the streaming function and similarity quantities of the form:

6 $$\begin{aligned} & A_{1} = \frac{\partial \psi }{{\partial y}},\;{\text{and}}\;A_{2} = - \frac{\partial \psi }{{\partial x}}, \\ & {\text{and}} \\ & \xi \left( {x,y} \right) = \sqrt {\frac{b}{{\upsilon_{f} }}} y,\;\theta \left( \xi \right) = \frac{{{\Theta } - {\Theta }}}{{{\Theta }_{w} - {\Theta }_{\infty } }},\;\psi \left( {x,y} \right) = \sqrt {\upsilon_{f} b} xf\left( \xi \right), \\ \end{aligned}$$

By replacing Eq. (6) into the system of Eqs. (1)–(3), the following outcome is obtained:

7 $$f^{{{\prime }2}} - 2{\Lambda }_{M} ff^{{\prime }} f^{{\prime \prime }} + \frac{1}{{\phi_{a} \phi_{b} }}\left( {K_{M} f^{{\prime }} - f^{{{\prime \prime \prime }}} } \right) - ff^{{\prime \prime }} + {\Lambda }_{N} f^{2} f^{{{\prime \prime \prime }}} - \frac{{\phi_{c} }}{{\phi_{b} }}Mf^{{\prime }} = 0,$$

8 $$\theta^{{\prime \prime }} \left( {1 + \frac{1}{{\phi_{e} }}PrR_{M} } \right) + \frac{{PrQ_{M} }}{{\phi_{e} }}\theta + \frac{PrEc}{{\phi_{a} \phi_{e} }}f^{{{\prime \prime }2}} + \frac{{\phi_{c} M\;Pr\;Ec}}{{\phi_{e} }}f^{{{\prime }2}} + \frac{{\phi_{d} \delta_{M} Pr}}{{\phi_{a} }}\left( {ff^{{\prime }} \theta^{{\prime }} + f^{2} \theta^{2} - f^{{{\prime }2}} \theta } \right)$$

With

9 $$\begin{aligned} f\left( 0 \right) & = S,\quad f^{{\prime }} \left( 0 \right) = 1 + \varpi_{N} f^{{\prime \prime }} \left( 0 \right),\quad f^{{\prime }} \left( \xi \right) \to 0,\quad \theta^{{\prime }} \left( 0 \right) = - B_{i} \left( {1 - \theta \left( 0 \right)} \right), \\ & \quad \theta \left( \xi \right) \to 0\;{\text{as}}\;\gamma \to \infty . \\ \end{aligned}$$

where

$$\begin{aligned} Q_{M} & = \frac{{Q_{0} }}{{\left( {\rho C_{p} } \right)_{f} b}}, \alpha_{f } = \frac{{k_{f} }}{{\left( {\rho C_{p} } \right)_{hnf} }},\varpi_{M} = \sqrt {\frac{b}{{\mu_{f} }}} P_{w} , B_{i} = \frac{{h_{f} }}{{k_{o} }}\sqrt {\frac{{\upsilon_{f} }}{b}} ,\delta_{M} = b\omega , \\ R_{M} & = \frac{16}{3}\frac{{\sigma^{*} T_{\infty }^{3} }}{{k^{*} \upsilon_{f} \left( {\rho C_{p} } \right)_{f} }},{\Lambda }_{M} = b\varsigma , \\ K_{M} & = \frac{{\upsilon_{f} }}{bk},\phi_{a} = \left( {1 - (\phi_{1} + \phi_{2} )} \right)^{2.5} , \phi_{b} = \left( {1 - (\phi_{1} + \phi_{2} )} \right) + \phi_{1} \rho_{{s_{1} }} /\rho_{f} + \phi_{2} \rho_{{s_{2} }} /\rho_{f} , \\ \phi_{c} & = \left( {1 - (\phi_{1} + \phi_{2} )} \right) + \phi_{1} \left( {\rho C_{p} } \right)_{{{\text{s}}1}} /\left( {\rho C_{p} } \right)_{f} + \phi_{2} \left( {\rho C_{p} } \right)_{s2} /\left( {\rho C_{p} } \right)_{f} , \\ \phi_{d} & = \left( {\frac{{(k_{{{\text{s}}2}} + 2k_{nf} - 2\phi_{2} \left( {k_{nf} - k_{{{\text{s}}2}} } \right)}}{{(k_{{{\text{s}}2}} + 2k_{nf} + \phi_{2} \left( {k_{nf} - k_{{{\text{s}}2}} } \right)}}} \right) \times \left( {\frac{{k_{{{\text{s}}1}} + 2k_{f} - 2\phi_{1} \left( {k_{f} - k_{{{\text{s}}1}} } \right)}}{{(k_{{{\text{s}}1}} + 2k_{f} + \phi_{1} \left( {k_{f} - k_{{{\text{s}}1}} } \right)}}} \right). \\ \end{aligned}$$

The formula (1) is satisfied identically. The notation ' denotes differentiation to ($\xi$), as shown in the equations above. The thermophysical properties of the nanofluid, along with the symbols used in the study, are outlined in Table 2 located below.

**Table 2:** Display the symbols and description in the governing equation.

${\mu }_{hnf}$	Dynamic viscosity	Pr	Prandtl number
${R}_{M}$	Solar radiation parameter	${E}_{N}$	Eckert number
${\alpha }_{f}$	Thermal diffusivity	$\varsigma$	Fluid relaxation
${\delta }_{M}$	Relaxation time	${K}_{M}$	Porous medium
${Q}_{M}$	Heat source	${k}_{g}$	Heat transport factor
${\sigma }_{hnf}$	Electrical conductivity	${R}_{\mathrm{w}}$	Porous stretchable surface
$\Theta$	Temperature	$\rho {C}_{p}$	Specific heat
${k}_{hnf}$	Thermal conductance	${\Lambda }_{N}$	Non-Newtonian Maxwell
${\mathrm{Re}}_{x}$	Local Reynolds number	${P}_{w}$	velocity slip
${\Lambda }_{N}$	Deborah number	$\beta$	Dimensionless temperature gradient
${Q}_{N}$	Heat generation	$S$	Suction/injection parameter
${\varpi }_{M}$	Velocity slip	${B}_{i}$	Biot number
${C}_{f}$	Drag force	Re	Reynolds number
${\phi }_{a},{\phi }_{b},{\phi }_{c},{\phi }_{d}$	Thermodynamics physical variables of Maxwell HNF	${N}_{G}$	Entropy generation
${\mathrm{Nu}}_{x}$	Nusselt number	${B}_{N}$	Brinkmann number

### Engineering quantities of interests

Some branches of engineering rely on two important parameters: drag force ($${C}_{f})$$ and Nusselt number ($${\mathrm{Nu}}_{x}).$$ The drag force measures how much resistance a solid object experiences while moving through a fluid, which is crucial in fields such as aerospace and fluid mechanics^82, 83^. Meanwhile, the Nusselt number predicts how quickly heat transfers between a liquid and a solid surface, which is important in the design of heat exchangers. Both parameters are unitless and are used to improve the efficiency of engineering designs. The $${C}_{f}$$ together with $${\mathrm{Nu}}_{x}$$ can be stated as^84^:

10 $$\begin{aligned} & C_{f} = \frac{{\tau_{w} }}{{\rho_{hnf} U_{w}^{2} }}, \\ & {\text{and}} \\ & {\text{Nu}}_{x} = \frac{{xq_{w} }}{{k_{f} \left( {{\Theta }_{w} - {\Theta }_{\infty } } \right)}}, \\ \end{aligned}$$

By applying dimensionless conversions to the equation mentioned earlier, we obtain:

11 $$Re_{x}^{1/2} C_{f } = \frac{1}{{\phi_{a} }}f^{{\prime \prime }} (0),\;{\text{and}}\;{\text{Re}}_{x}^{1/2} \;{\text{Nu}}_{x} = - \frac{{k_{hnf} }}{{k_{f} }}\left[ {\left( {1 + R_{N} } \right)\theta^{\prime}\left( 0 \right)} \right]$$

Here, $${\mathrm{Re}}_{x}$$=$$\frac{{U}_{w}x}{{v}_{f}}$$.

### Entropy generation

In the field of thermodynamics, the principle of entropy generation describes the gradual reduction in a system's ability to perform work as energy becomes less available^85^. This principle is especially important to solar radiation, as it provides a means of measuring the inefficiencies that arise during the conversion of sunlight into usable forms of energy. Energy loss transpires due to the lack of efficiency exhibited by the solar panels and other components that are utilized to transform energy. To maximize the efficiency of solar energy conversion, it is important to minimize the amount of energy lost as entropy. This can be achieved by advanced materials and design techniques that reduce energy losses and increase the amount of usable energy obtained from solar radiation. The second law of thermodynamics offers a means to approximate the amount of irreversibility or entropy generation that occurs during the process of nanofluid flow. This can be mathematically expressed as^35^:

12 $${N}_{G}={\mathrm{Re}}\left({\phi }_{d}\left(1-\mathrm{Nr}\right){\theta }^{{^{\prime}}2}+\frac{{B}_{N}}{\beta }\frac{1}{{\phi }_{a}}\left({Kf}^{{^{\prime}}2}\right)+{\phi }_{a}{\phi }_{b}M{f}^{{^{\prime}}2}+\frac{{B}_{N}}{\beta }\frac{1}{{\phi }_{a}}\left({f}^{{^{\prime}}{^{\prime}}2}\right)\right),$$

where Re = $\frac{{u}_{w}{b}^{2}}{x{\upsilon }_{f}}$, $\beta =\frac{{\mathrm{T}}_{w}-{\mathrm{T}}_{\infty }}{{\mathrm{T}}_{\infty }}$ and,${B}_{N}=\frac{{\mu }_{f}{u}_{w}^{2}}{{k}_{f}\left({\mathrm{T}}_{w}-{\mathrm{T}}_{\infty }\right)}$

## Numerical outcome

These proposed solutions for the functions $f\left(\xi \right)$, $and \theta \left(\xi \right)$ are^76^:

13 $$f\left(\xi \right)={\sum }_{j=0}^{M}{U}_{j}{A}_{j}\left(\frac{2\eta }{L}-1\right), \mathrm{and} \theta \left(\xi \right)={\sum }_{j=0}^{M}{V}_{j}{A}_{j}\left(\frac{2\eta }{L}-1\right),$$

Here, ${A}_{j}\left(\frac{2\xi }{L}-1\right)$ is a shifted Legendre base function that is defined in the interval [1;1] to [0; L]. To solve for the values of constants ${a}_{j}$, and ${b}_{j}$ we need to use formula (13) and substitute it into the BCs.

$$\left({{\sum }_{j=0}^{M}{U}_{j}{A}_{j}\left(\frac{2\xi }{L}-1\right)}_{\xi =0}\right)=0$$

$$\left(\frac{d}{d\xi } {{\sum }_{j=0}^{M}{U}_{j}{A}_{j}\left(\frac{2\xi }{L}-1\right)}_{\xi =0}\right)=1+{\varpi }_{M}\left(\frac{{d}^{2}}{{d\xi }^{2}} {{\sum }_{j=0}^{M}{U}_{j}{A}_{j}\left(\frac{2\xi }{L}-1\right)}_{\xi =0}\right)$$

14 $$\left(\frac{d}{d\xi }{{\sum }_{j=0}^{M}{V}_{j}{A}_{j}\left(\frac{2\eta }{L}-1\right)}_{\xi =0}\right)=-{B}_{i}\left(1-{{\sum }_{j=0}^{M}{V}_{j}{A}_{j}\left(\frac{2\eta }{L}-1\right)}_{\xi =0}\right)$$

By inserting formula (13) into formula (7–8), we obtained three residuals: ${R}_{f}\left(\xi \right)$, and ${R}_{f}\left(\xi \right)$, The collocation method is utilized, and its description is provided as follows:

15 $${\text{For}}\;\psi \left( {\xi - \xi_{k} } \right) = \left\{ {\begin{array}{*{20}c} {1,} & {\xi = \xi_{j} } \\ {0,} & {{\text{otherwise}},} \\ \end{array} } \right.$$

with

16 $${\int }_{0}^{L}{R}_{f} \psi \left(\xi -{\xi }_{k}\right)d\xi ={R}_{f}\left({\xi }_{j}\right)=0, for j=1, 2,..N-2\mathrm{ and},{\int }_{0}^{L}{R}_{\uptheta } \psi \left(\xi -{\xi }_{k}\right)d\xi ={R}_{\theta }\left({\xi }_{j}\right)=0, for j=1, 2,..M-1$$

The shifted Gauss–Lobatto points are denoted as ${\xi }_{j}$. The system of algebraic Eqs. (7–8) includes 2N + 2 equations, and with coefficients that are unknown ${a}_{j}$, and ${b}_{j}$. These coefficients were calculated using the MATHEMATICA software. Table 3 displays the different degrees of approximation convergence. Nonetheless, LBCS yields a rapid convergence of approximate solutions. The validity of the numerical solution used in the current study is demonstrated by comparing the results with those obtained in a previous study. Table 4 presents this comparison, and it shows that there is a strong correlation between the numerical outcomes of both studies.

**Table 3:** Different approximation orders of convergence for LBCM solutions when ${\Lambda }_{M},{={E}_{M}=Q}_{M}$=$0.1$, ${K}_{M}={B}_{i}={R}_{M}$=1.4, $S$=0.2

Number of iteration (N)	${f}^{\mathrm{^{\prime}}}(\eta )$	ϕ (0)
4	$4.3549$	3.5351
6	$4.2449$	3.3229
8	$4.1524$	3.2932
10	$4.0001$	3.2309
12	$4.0001$	3.1192
14	$4.0001$	3.1192
16	$4.0001$	3.1192
20	$4.0001$	3.1192
24	$4.0001$	3.1192
30	$4.0001$	3.1192

**Table 4:** Presents a numerical comparison of the $N{u}_{x}$ for Prandtl number (Pr).

$\mathrm{Pr}$	Outcome of^86^	Outcome of^87^	Outcome of^88^	Outcome of^89^	Present outcome
0.72	0.80876181	0.8086	0.80876122	0.8086	0.80876181
1.0	1.00000000	1.0000	1.00000000	1.0000	1.00000000
3.0	1.92357420	1.9236	1.92357431	1.9237	1.92357420
7.0	3.07314651	3.0723	3.07314679	3.0723	3.07314651
10	3.72055429	3.7006	3.07314679	3.7207	3.72055429

Significant values are in bold.

## Result and discussion

**Figure 4:**
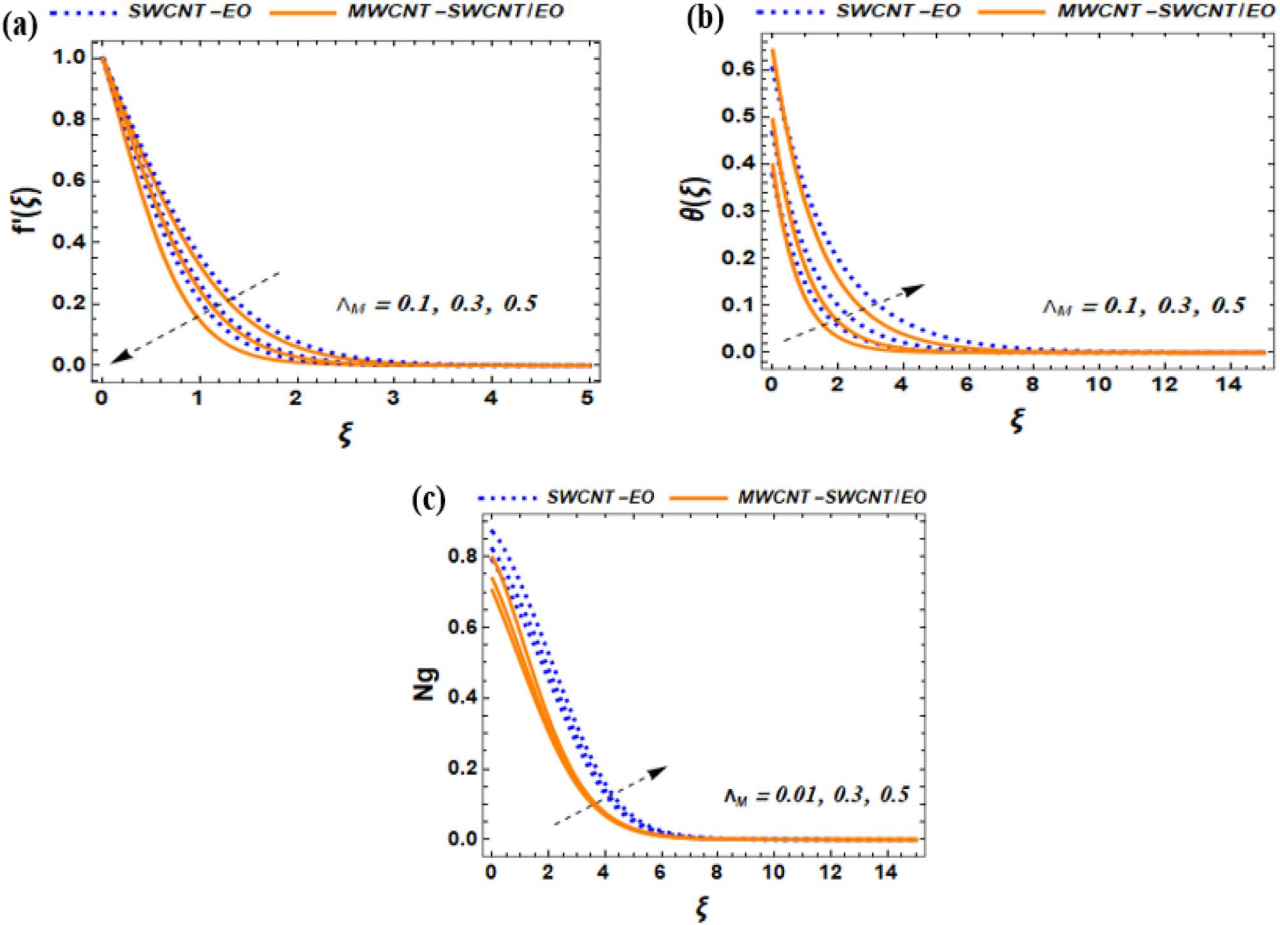
Effect of ${\Lambda }_{M}$ on ${f}^{^{\prime}}\left(\xi \right)$, $\theta \left(\xi \right)$ and ${N}_{G}$.

**Figure 5:**
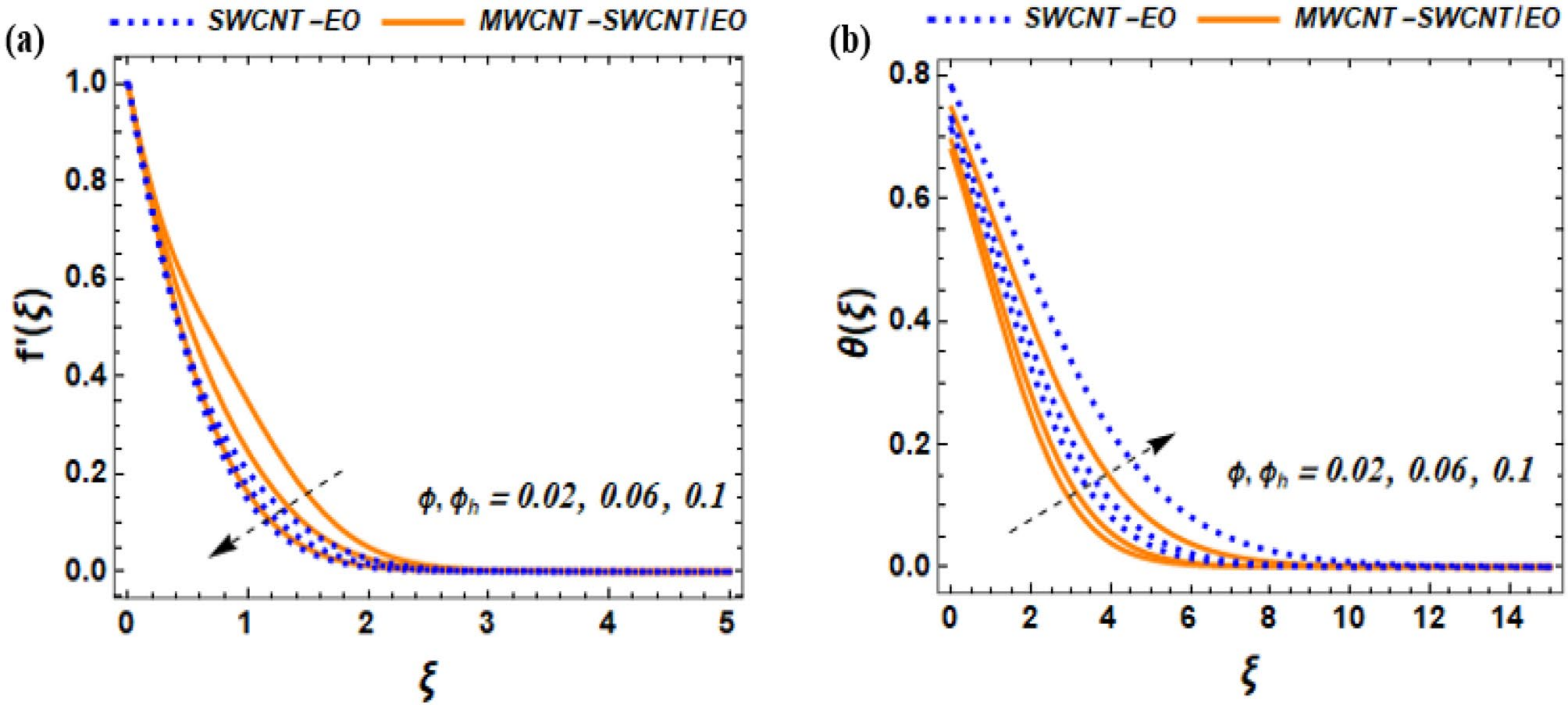
Effect of $\varphi {,\varphi }_{h}$ on ${f}^{^{\prime}}\left(\xi \right)$, and $\theta \left(\xi \right)$.

**Figure 6:**
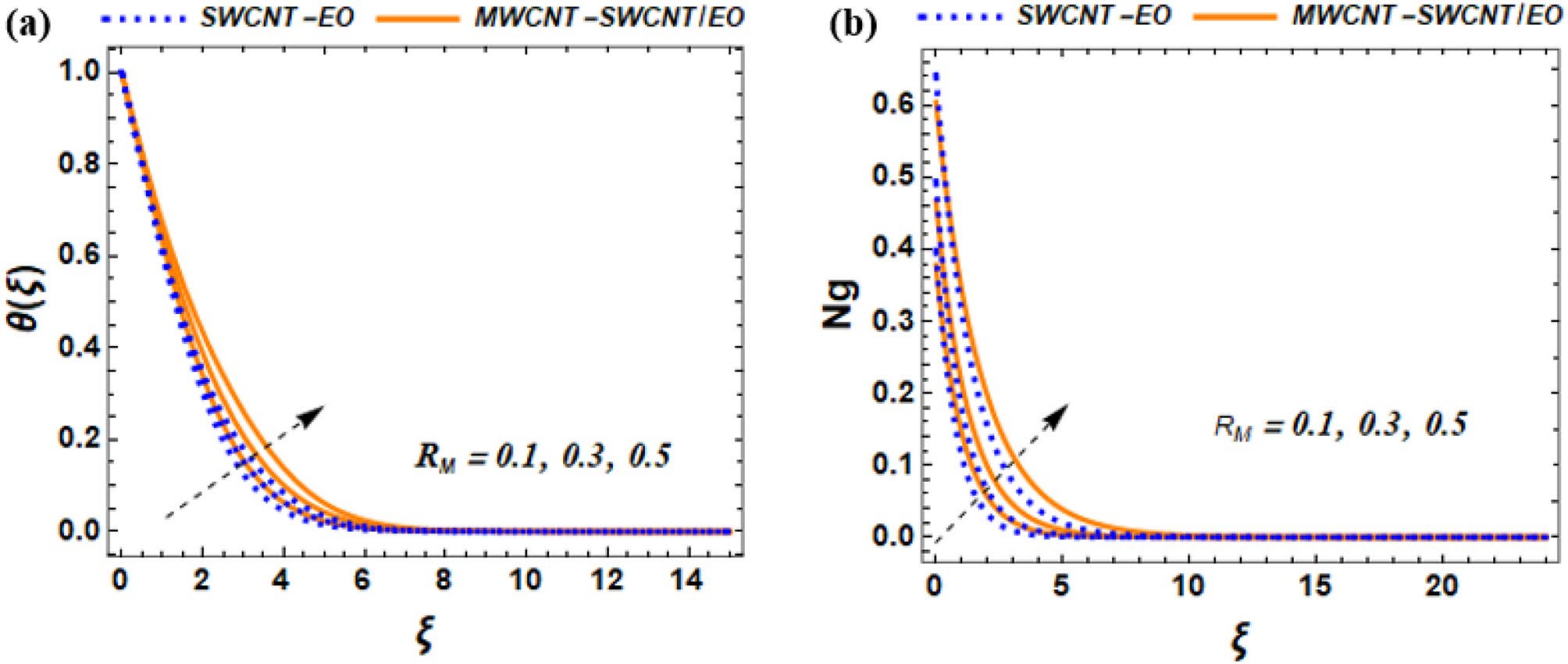
Effect of effect of ${R}_{M}$ on $\theta \left(\xi \right)$, and Ng.

**Figure 7:**
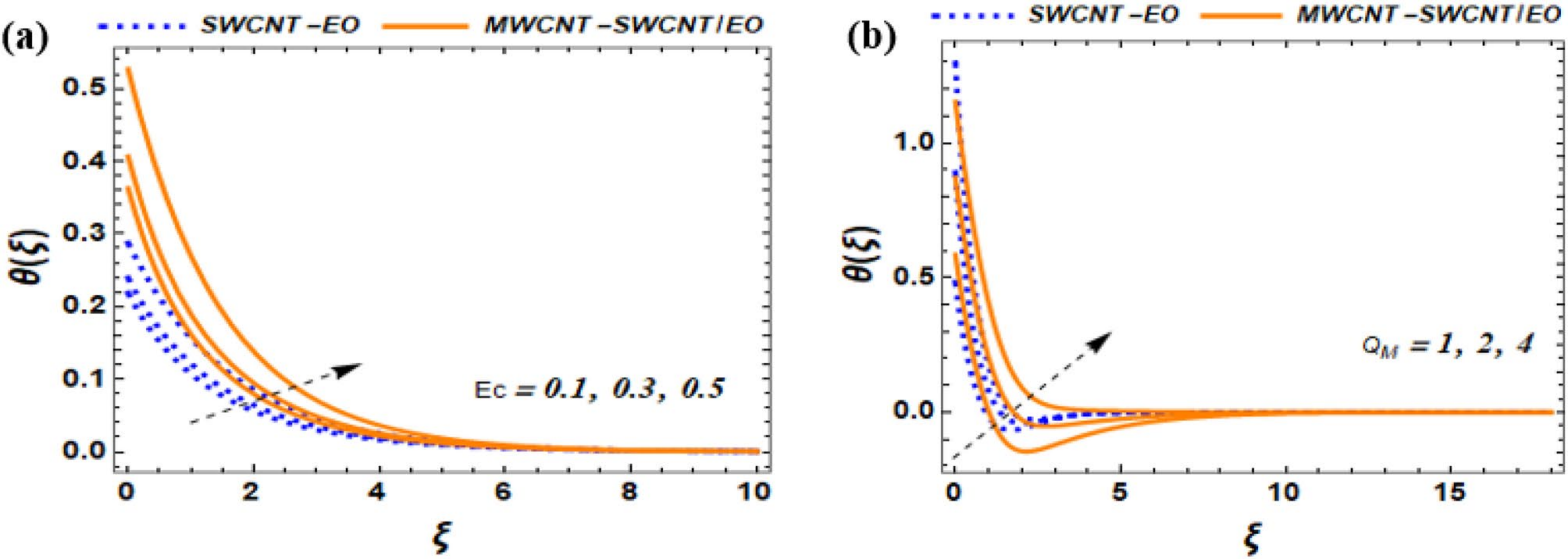
Impact Ec and ${Q}_{M}$ on $\theta \left(\xi \right)$.

**Figure 8:**
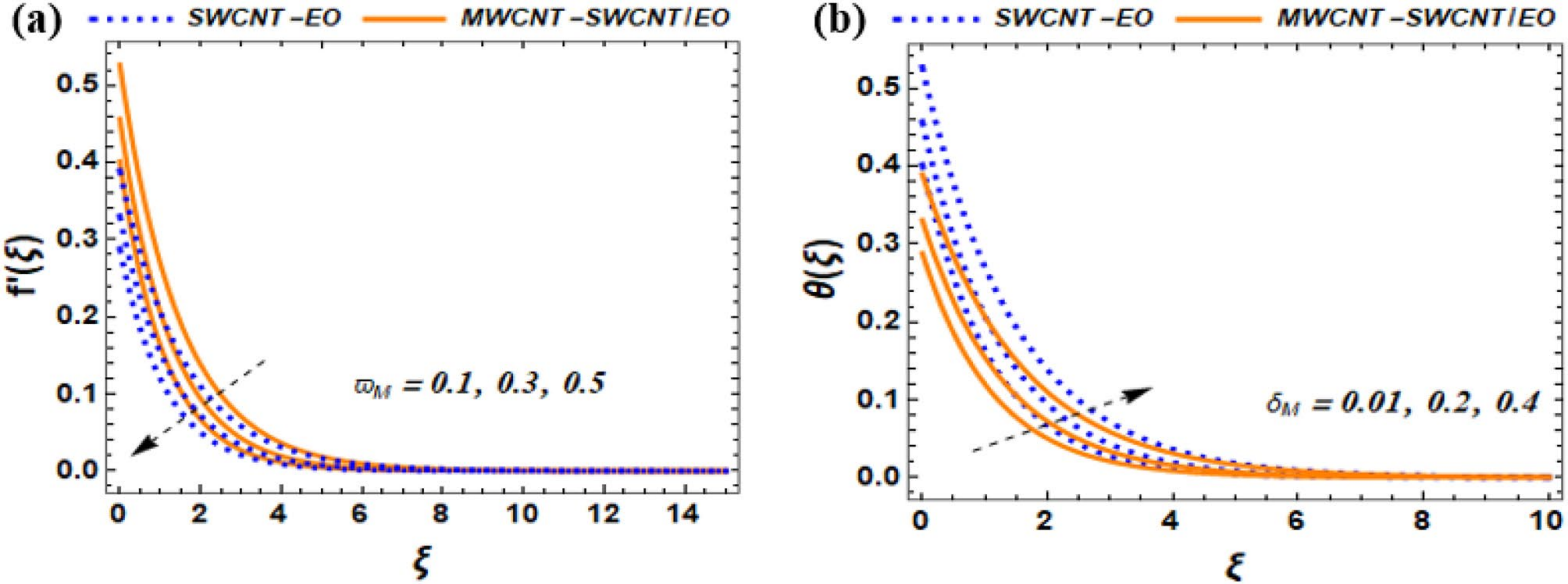
Impact ${\varpi }_{M}$ and ${\delta }_{N}$ ${f}^{^{\prime}}\left(\xi \right)$, and $\theta \left(\xi \right)$.

**Figure 9:**
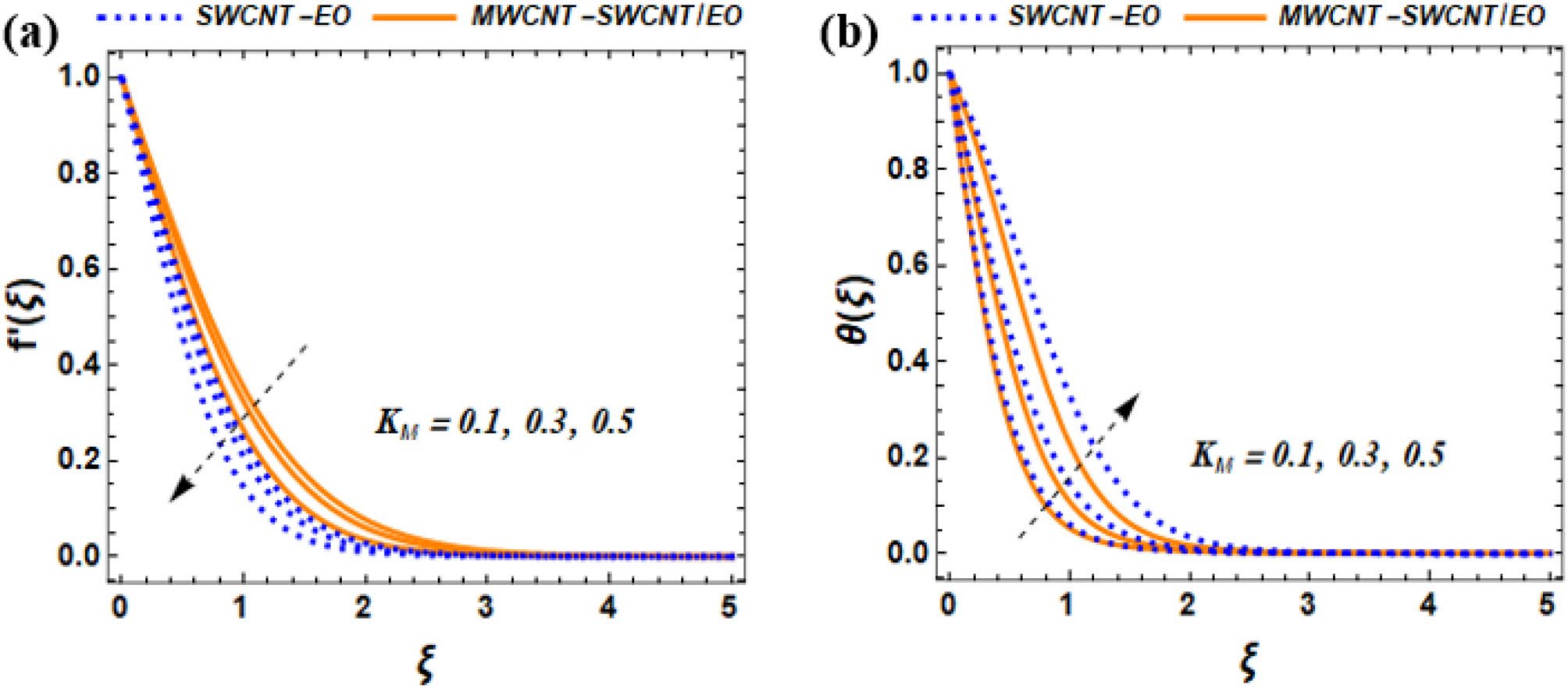
Effect of ${K}_{M}$ on $f{^{\prime}}(\xi ),and \theta (\xi )$.

**Figure 10:**
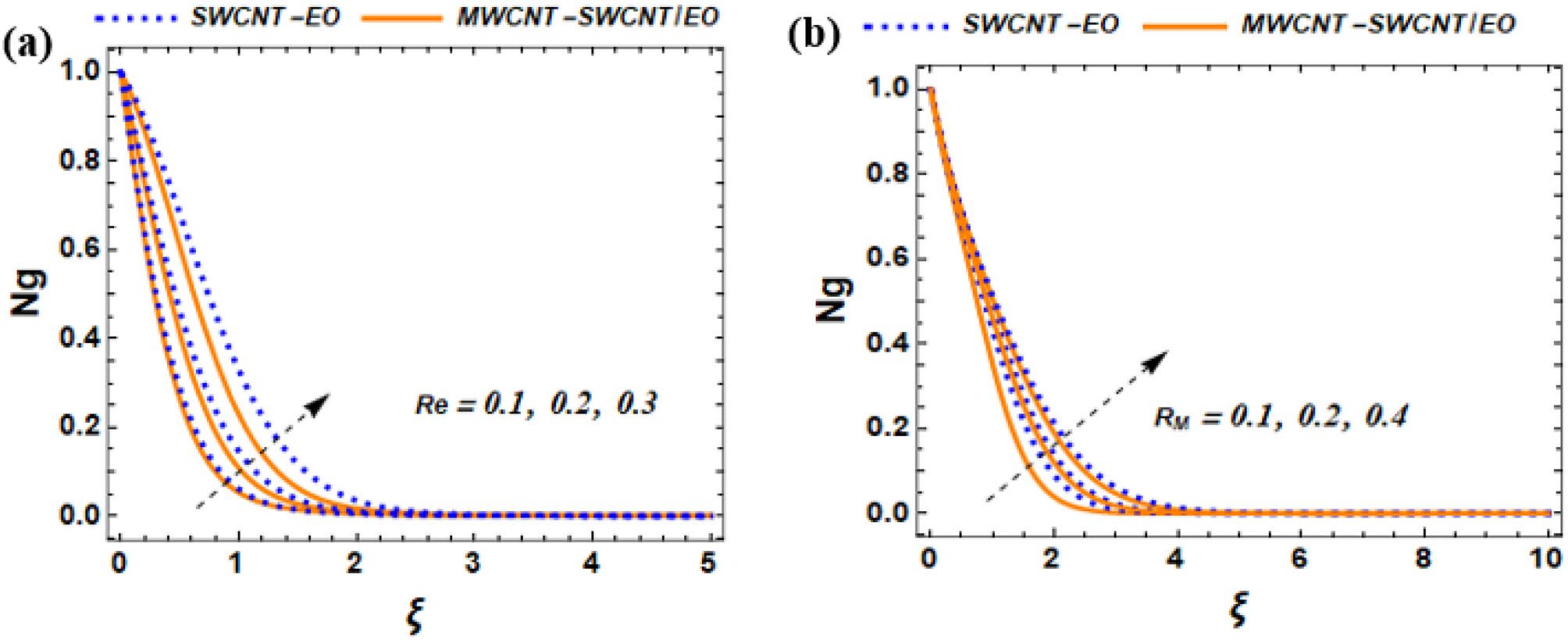
Impact of ${B}_{M},$ and Re on Ng.

This section of the study displays graphs that illustrate changes in fluid velocity $({\mathrm{f}}^{\prime}({\upxi })),\;{\text{and}}\;{\text{thermal}}\;{\text{profile}}\;({{\theta (\xi )}})\;{\text{entropy}}\;{\text{production}}\;{\text{(Ng)}}$ for MWCNT-SWCNT/EO hybrid nanofluid (HNF) and MWCNT –SWCNT nanofluid (NF). Figures 3a, 4a, 5a, 8a, 9a show variations in $\mathrm{f}{{^{\prime}}}(\upxi )$, while Figs. 3b, 4b, 5b, 6a,b, 7a,b, 8b, 9b depict changes in $\uptheta (\upxi )$. Additionally, Figs. 4c, 10a,b shows variations in $(\mathrm{Ng})$. Furthermore, the study presents the numerical results of the Nusselt number. The results are shown in the form table (Table 5).

**Table 5:** Calculation of ${\mathrm{Nu}}_{x}$ for Pr = 6450.

Ec	${R}_{M}$	${\delta }_{M}$	$\mathrm{M}$	${B}_{i}$	${\mathrm{Nu}}_{x}$ SWCNT-EO	${\mathrm{Nu}}_{x}$ MWCNT-SWCNT/EO	Relative $\frac{{\mathrm{Nu}}_{\mathrm{MWCNT}-\mathrm{SWCNT}/\mathrm{EO}}-{\mathrm{Nu}}_{\mathrm{SWCNT}/\mathrm{EO}}}{{\mathrm{Nu}}_{\mathrm{MWCNT}}}$ $\times 100$
2	0.1	0.1	0.2	0.1	5.6135	5.7234	1.96%
4	0.1	0.1	0.2	0.1	5.4642	4.5799	2.07%
6	0.1	0.1	0.2	0.1	5.4159	5.5339	2.13%
2	0.1	0.1	0.2	0.1	5.5141	5.5251	1.99%
2	0.3	0.1	0.2	0.1	5.5199	5.6642	2.05%
2	0.5	0.1	0.2	0.1	5.6850	5.8159	2.25%
2	0.1	0.1	0.2	0.1	5.5749	5.6789	1.82%
2	0.1	0.2	0.2	0.1	5.6141	5.7229	1.90%
2	0.1	0.4	0.2	0.1	5.6591	5.7761	2.05%
2	0.1	0.1	0.1	0.1	5.3527	5.5067	2.02%
2	0.1	0.1	0.2	0.1	5.3721	5.5191	2.06%
2	0.1	0.1	0.3	0.1	5.5271	5.5300	2.07%
2	0.1	0.1	0.2	0.1	5.5681	5.6692	1.78%
2	0.1	0.1	0.2	0.2	5.6136	5.7247	1.94%
2	0.1	0.1	0.2	0.4	5.6599	5.7913	2.26%

### Parametrical study on relative thermal transport rate

Understanding the Nusselt number is crucial to fully comprehending the thermal transfer rate. It is one of the key factors that play a significant role in determining the outcomes of the thermal transfer process. The Nusselt numbers in Table 5 serve as evidence that the use of MWCNT-SWCNT/EO hybrid nanofluid (HNF) combinations lead to a higher thermal transfer rate compared to the MWCNT-SWCNT nanofluid (NF). This validates the expectation that the HNF combinations would result in a greater thermal transfer rate. Table 5 displays a breakdown of percentages, which reveals that an increase in the Ec value resulted in a noticeable increase. The smallest percentage observed was 1.96%, while the largest was 2.13%. The table shows an increase in ${R}_{M}$. The percentage difference between the smallest point and the greatest point is 1.99% and 2.25%, respectively. In addition, the study demonstrated that the minimum and maximum values for the relative HNF and NF occur between 2.02 and 2.07%as the value of ${\delta }_{M}$ increases. As the value of $\mathrm{M}$ increases, the range of values observed is between 2.02 and 2.07%, with 2.2% being the minimum value and 2.7% being the maximum value. The thermal transfer rate of HNF and NF increases by a minimum of 2.2–2.6% as the value of ${B}_{i}$ increases. This means that all the physical parameters contribute positively to the rate of thermal transfer.

### Impact of magnetic parameter $M$ on $f^{\prime}\left( \xi \right),{\text{and}}\;\theta \left( \xi \right)$

The effect of M on the velocity profile is seen in Fig. 10. As the strength of the magnetic field intensifies, it exerts a greater force on the charged particles present in the fluid, leading to an upsurge in resistance. This rise in resistance consequently leads to a further reduction in the velocity of the fluid. Boundary layer flow pertains to the thin layer of fluid adjacent to a surface, which may experience instability and shift from a smooth and orderly flow to a disordered and turbulent flow. Such a shift can bring about an elevation in drag force experienced by objects moving through the fluid, ultimately leading to reduced efficiency. Based on the presence of hybrid nanoparticles in the base fluid, the hybrid nanofluid appears to show some decline compared to the regular nanofluid at the same point. The regular NF makes it more difficult for the magnetic field to influence the fluid, which could explain this slight performance improvement. Figure 3b illustrates the phenomenon where the temperature of the system rises as a result of reduced heat transfer to other areas caused by the slower movement of the fluid flow. If the heat transfer coefficient value is positive, it indicates that heat is moving from the surface to the fluid. This means that the surface is releasing heat energy while the fluid is absorbing it. Understanding this mechanism is critical for numerous engineering applications, including cooling systems for electronics or engines, and it can help improve the systems' effectiveness.

### Impact of Maxwell parameter ${{\varvec{\Lambda}}}_{{\varvec{N}}}$ on ${\varvec{f}}\mathbf{^{\prime}}({\varvec{\upxi}}),{\varvec{\theta}}({\varvec{\upxi}}),\mathbf{a}\mathbf{n}\mathbf{d}\mathbf{N}\mathbf{g}$

The Deborah number is a mathematical concept that defines how the elasticity and viscosity of a fluid relate to one another. It plays a crucial role in determining how a fluid responds to different kinds of pressure and force. From Fig. 4a, it was observed that the velocity profile of a fluid is reduced by ${\Lambda }_{M}$. Meanwhile, when Deborah number is low, the fluid becomes more elastic, and its velocity profile becomes parabolic. This occurs because elastic forces are more dominant than viscous forces, causing the fluid to behave like a solid. while the lowest velocity occurs near the walls, creating a curved parabolic shape. As the ${\Lambda }_{M}$ increases, the fluid's ability to flow changes. The higher the ${\Lambda }_{M}$ increases, the more viscous the fluid becomes and the less elastic it becomes. Also, the fluid behaves more like a normal liquid, with viscous forces being dominant over elastic forces. As observed in Fig. 4a, the velocity flow of the Maxwell hybrid nanofluid exhibits a decreasing pattern. Figure 4b illustrates the impact of ${\Lambda }_{M}$ on the temperature profile. In the case of low Deborah numbers, the fluid exhibits Newtonian behavior, where the influence of viscous forces dominates over elastic forces. When the ${\Lambda }_{M}$ rises, the elastic forces have a greater impact than the viscous forces, which leads to the creation of temperature variations in the fluid. This phenomenon arises because the elastic forces induce deformation and relaxation patterns in the fluid. The experiences a greater improvement in temperature distribution compared to the hybrid nanofluid. From Fig. 4c, As the Deborah number rises, so does the entropy generation increase. This phenomenon can be attributed to the fact that higher Deborah numbers are linked to greater shear rates and longer relaxation times, which cause a more significant amount of irreversible energy loss.

### Impact of volume fraction of nanoparticles ***(***${\varvec{\phi}}$***)*** on $f^{\prime}\left( \xi \right),\;{\text{and}}\;\theta \left( \xi \right)$

The behavior of fluids, such as how they flow and distribute heat, is significantly impacted by the presence of nanoparticles in the fluid (see Fig. 5a,b). The friction created by these nanoparticles plays a crucial role in determining the physical characteristics of the fluid. Additionally, the size of the nanoparticles is an important factor that has five specific quantities, which are denoted by i.e. ${\phi }_{a}$=${\left(1-{(\phi }_{1}{+\phi }_{2})\right)}^{2.5} , {\phi }_{b}$=$\left(1-{(\phi }_{1}{+\phi }_{2})\right)+{\phi }_{1}{\rho }_{{s}_{1}}/{{\rho }_{f}+\phi }_{2}{\rho }_{{s}_{2}}/{\rho }_{f}$,${\phi }_{c}=\left(1-{(\phi }_{1}{+\phi }_{2})\right)+{\phi }_{1}{\left(\rho {C}_{p}\right)}_{\mathrm{s}1}/{\left(\rho {C}_{p}\right)}_{f}$+${\phi }_{2}{\left(\rho {C}_{p}\right)}_{s2}/{\left(\rho {C}_{p}\right)}_{f}, {\phi }_{d}$=$\left(\frac{({k}_{\mathrm{s}2}+2{k}_{nf}-{2\phi }_{2}\left({k}_{nf}-{k}_{\mathrm{s}2}\right)}{({k}_{\mathrm{s}2}+2{k}_{nf}+{\phi }_{2}\left({k}_{nf}-{k}_{\mathrm{s}2}\right)}\right)\times \left(\frac{{k}_{\mathrm{s}1}+2{k}_{f}-2{\phi }_{1}\left({k}_{f}-{k}_{\mathrm{s}1}\right)}{({k}_{\mathrm{s}1}+2{k}_{f}+{\phi }_{1}\left({k}_{f}-{k}_{\mathrm{s}1}\right)}\right)$, based on the Tiwari–Das nanoscale model. However, the behavior of the flow at the boundary layer (BL) in the presence of solar radiation can be altered by the presence of nanoparticles in a fluid. The BL of fluid that is in direct contact with a surface, such as a solar panel, is known as the boundary layer, and it is influenced by the heat transfer from solar radiation. Changing the proportion of nanoparticles in the fluid can cause a modification in the velocity profile of the boundary layer flow. As the nanoparticles’ fractional volume parameter increases, the fluid flow near the solar panel’s surface becomes more uniform and less turbulent. This leads to a decrease in the fluid’s velocity at the solar panel’s surface. Therefore, increasing the nanoparticle concentration causes a reduction in the fluid velocity (see Fig. 5a). The movement of fluids and particles in a system is greatly influenced by two important factors. The first factor is the nanoparticle fractional volume parameter, which has a significant impact on the flow behavior of fluid-particle systems. The second factor is the temperature profile of the system, which describes the distribution of temperature among the fluid and particles. These two factors are critical in understanding the behavior of fluid-particle systems. The concentration of nanoparticles in fluid-particle systems has a significant impact on temperature distribution. As nanoparticle concentration increases, temperature distribution becomes more diverse due to differences in the thermal properties of the fluid and particles. Higher nanoparticle concentration results in a more uniform temperature distribution because heat transfer between the fluid and particles is more effective. This suggests that nanoparticle concentration is an important factor in controlling temperature distribution in fluid-particle systems (see Fig. 5b).

### Influence of radiative heat flux parameter (${R}_{M}$) on ${f}^{^{\prime}}\left(\xi \right)$, and $\theta \left(\xi \right)$

The radiative heat flux parameter is essential in determining how solar radiation impacts the temperature distribution of a system. This radiative heat flux parameter is responsible for the transfer of thermal energy through radiation and determines how much energy a surface will absorb or reflect. Its value is significant as it directly affects the temperature profile of the system. The influence of ${\mathrm{R}}_{\mathrm{M}}$ on thermal distribution is display in Fig. 6a. As observed in Fig. 6a, the thermal distribution for the radiative heat flux of hybrid nanofluid exhibits a increasing pattern. However, as more solar radiation is absorbed by the surface, it causes a increasement in its temperature due to the additional energy received. Physically, the reason behind this occurrence is that the sun's energy, which is also referred to as solar radiation, increases the surface temperature by providing energy. Also, this temperature rise can be further amplified by enhancing the radiative heat flux parameter, allowing the surface to absorb more solar energy. The main factor responsible for this effect is the extra energy input from the sun, which leads to a temperature rise. The implications of this phenomenon are noteworthy in different areas, such as solar energy, engineering, and materials science. The influence of ${\mathrm{R}}_{\mathrm{M}}$ on entropy generation is display in Fig. 6b. The radiative heat flux parameter has a significant effect on how entropy generation, remarkably in systems that are exposed to solar radiation. However, this means that understanding the impact of radiative heat flux parameter is crucial in accurately modeling and predicting entropy generation in these systems. The entropy generation rate can be used to measure the degree of irreversibility in each process. As ${\mathrm{R}}_{\mathrm{M}}$ is increasing, the rate at which entropy is generated will increase. Physically, this occurs because the increased radiative heat flux causes a greater temperature difference between the system and its surroundings, which leads to more heat transfer and more entropy generation. To create energy technologies that are environmentally sustainable and efficient, it is necessary to understand the underlying physical mechanisms and minimization of entropy generation. This can lead to the development of new technologies.

### Influence of Eckert number (Ec) and heat generation ${(Q}_{M})$ on $f^{{\prime }} \left( \xi \right),{\text{and}}\;\theta \left( \xi \right)$

The Eckert number (Ec) is providing insights into the proportion of kinetic energy to thermal energy in a fluid flow. Specifically, it indicates the ratio between the density of kinetic energy and the density of thermal energy, which helps in understanding the balance between these two types of energy present in the flow. To comprehend the impact of the Eckert number on the temperature of fluid, it is essential to analyze the equilibrium between the kinetic and thermal energy present within the flow. The influence of Ec on thermal distribution is display in Fig. 7a. A high Eckert number indicates a dominance of kinetic energy over thermal energy, which results in the flow being controlled by kinetic energy. When the Eckert number is low, it indicates that the thermal energy in the fluid is much greater than its kinetic energy. This implies that the fluid's flow is mainly influenced by its thermal energy. Consequently, there will be noticeable temperature differences throughout the flow, which can result in variations in the temperature profiles. The reason behind this behavior can be understood by examining how solar radiation warms up the fluid. When solar radiation is absorbed, it heats up the fluid and causes it to expand, which results in the fluid having more energy in motion. This energy is then dissipated as the fluid moves through the flow due to viscous forces, ultimately transforming into heat energy. The Eckert number plays a crucial role in determining how fast this transformation takes place by controlling the balance between the energy of motion and heat energy in the flow. The Fig. 7b indicates that as the ${Q}_{M}$ is increased, it will be produced at a faster rate than it can be dissipated, causing the fluid to become hotter. This, in turn, leads to larger temperature differences within the fluid flow. This phenomenon is caused by how solar radiation interacts with the fluid. The fluid absorbs solar radiation, which increases its temperature and generates thermal energy. As observed in Fig. 7b, the thermal distribution for the radiative heat flux of hybrid nanofluid exhibits an increasing pattern of the regular nanofluid. Physically, if the rate of heat generation is too high, the thermal energy can accumulate faster than it can be released by the fluid, causing it to become hotter. This leads to greater temperature differences within the fluid flow.

### Influence of slip velocity $\left({\varpi }_{\mathrm{M}}\right)$ and relaxation time parameter ${(\delta }_{M})$*on *$f{^{\prime}}(\xi ),and \theta (\xi )$

The Influence of slip velocity $\left({\varpi }_{M}\right)$ increases the velocity profile (see Fig. 8a). It was shown that the velocity fluid temperature for hybrid nanofluid is higher than the nanofluid. The velocity slip is a term used to describe the difference in speed between the solid surface and the fluid in nanofluids, which is caused by the existence of nanoparticles in the fluid. This phenomenon has noteworthy effects on the behavior and characteristics of nanofluids and hence should be taken into account while dealing with the applications of these fluids. The velocity profile in nanofluids is influenced by the ${\mathrm{K}}_{\mathrm{M}}$, which depends on the unique characteristics of the nanoparticles (CNTs) and the base fluid (engine oil). As MWCNT-SWCNT is added to engine oil, the slip parameter causes a decrease in velocity near solid surfaces. Physically, the occurrence can be explained by the fact that CNTs have a hydrophobic surface, which causes a slippery effect where the solid and liquid phases meet. As the slip parameter increases, the fluid flow close to the surface becomes more uniform, which implies a weaker rate of change in velocity. The slip parameter of velocity has a noteworthy effect on the fluid velocity for a hybrid nanofluid, which leads to a reduction of the velocity gradient near solid surfaces. This phenomenon occurs due to the hydrophobic nature of carbon nanotubes, causing a slip condition at the interface of the liquid and solid. To put it simply, the slip parameter of velocity influences the way fluids flow around solid surfaces in nanofluids that contain carbon nanotubes due to their hydrophobic properties. The physical behavior of the relaxation time parameter ${\delta }_{M}$ is exhibited graphically in Fig. 8b. The relaxation time parameter denotes the duration that a fluid needs to spread out its thermal energy. This parameter is affected by the fluid's thermal conductivity as well as the properties of the suspended particles. When the fluid has higher thermal conductivity, the ${\delta }_{M}$ usually tends to be lower. In simpler terms, this parameter signifies the rate at which a fluid releases its heat energy, which depends on the fluid's heat-conducting ability and the size and shape of its particles. Hence, a fluid that can conduct heat more efficiently will have a shorter relaxation time. The use of a hybrid nanofluid containing both MWCNT-SWCNT/engine oil demonstrates superior heat conduction ability compared to SWCNT-engine oil.

### Influence of porous media parameter ${K}_{M}$ on $f^{{\prime}}(\xi ),and \theta (\xi )$

The porous media parameter ${\mathrm{K}}_{\mathrm{M}}$ hybrid nanofluid flow show how smoothly a fluid can enter a porous material, the fluid velocity distribution for mono is lower than the hybrid nanofluid. Furthermore, Darcy’s law explains how the movement of fluid is influenced by a porous material, specifically how its velocity is affected. The law essentially states that the fluid's flow through the porous medium is determined by the pressure gradient, the permeability of the medium, and the viscosity of the fluid. From Fig. 9a, it was observed that the as the fluid moves through a medium, it experiences resistance that leads to a decrease in velocity. However. The surface area of CNTs possesses the ability to enhance the process of heat transfer and the thermal conductivity of fluids. When these nanotubes are mixed with engine oil, they produce a fresh category of fluids known as NF. These NF have the potential to be effectively employed in various types of engineering projects. The effect of ${\mathrm{K}}_{\mathrm{M}}$ is displayed in Fig. 9a. It was observed the ${\mathrm{K}}_{\mathrm{M}}$ enhance the temperature profile. Physically, when sunlight enters the porous material, it is absorbed by the material and increases its temperature of the material. The significance of this phenomenon is emphasized in the context of the solar-powered ships, where the porous material serves as an effective tool to manage the temperature of the energy storage system, such as fuel cells or batteries. By maintaining the optimal temperature within these systems, it becomes possible to enhance their performance and longevity, resulting in a more dependable and eco-friendly operation of the ship.

### Influence of Brinkmann number ${(B}_{M}),$ and Reynolds number (Re) $\mathrm{on Ng}$

The Brinkman number is a dimensionless value that is used in the field of fluid mechanics to determine the importance of the viscous forces compared to the inertia forces within a fluid. It helps us understand how much the fluid's viscosity affects its behavior to its mass and movement. From Fig. 10a, the ${\mathrm{B}}_{\mathrm{M}}$ increases, there is a corresponding increase in the rate of entropy generation. Physically, the implementation of solar power technology in ships has the potential to lower the dependence on non-renewable energy sources and minimize the negative environmental effects of the shipping industry. The efficacy of solar energy is reliant on a multitude of factors, such as the accessibility of sunlight and the capacity of the photovoltaic cells. From Fig. 10a, as the Re increases, the rate of entropy generation rises due to the presence of a greater hybrid nanofluid in the boundary layer, which causes more energy to be dissipated in the form of heat. Physically, the optimization of solar panel efficiency and the creation of entropy in a solar-powered ship can be achieved by regulating the Reynolds number within the boundary layer of the system.

## Conclusion

In conclusion, this computational study has demonstrated the potential of using MWCNT-SWCNT/EO hybrid nanofluid (HNF) and MWCNT-SWCNT nanofluid (NF) and the Cattaneo–Christov heat flux model to improve the heat transfer performance of solar-powered ships. The results of this study highlight the promising prospects of this novel approach in achieving significant enhancements in heat transfer rates. With the increasing demand for renewable and sustainable energy sources, this approach provides a practical and efficient solution to overcome the challenges faced by solar-powered ships. The implementation of CNT hybrid nanofluids and the Cattaneo–Christov heat flux model is expected to contribute towards the development of more efficient and sustainable energy systems. Overall, this study provides a strong foundation for future research on the use of CNT hybrid nanofluids and the Cattaneo–Christov heat flux model to improve the performance of solar-powered ships.

- The outcome shows that the HNF has better thermal radiative enhancement compared to NF.
- By increasing the values of the ${R}_{M}$, ${K}_{M}$, ${\delta }_{M},$ it was observed that the velocity field decreases.
- The parameter ${\varpi }_{M}$ has a negative impact on the fluid velocity, causing it to decrease as ${\varpi }_{M}$ increases.
- As nanomaterials are present along with ${R}_{M}$, Ec, and ${Q}_{M}$ the thickness of the thermal boundary layer gradually increases over time, leading to a decrease in the rate of heat exchange.
- The entropy generation is influenced by various parameters, such as the ${R}_{M},$ ${B}_{M},$ Re, and ${\Lambda }_{M}$.
- The thermal efficiency of HNF performs better than NF, with a relative improvement ranging from 1.78 to 2.25%.
- The increasing values of ${\delta }_{M}$ ${\Lambda }_{N}$ ${K}_{M}$, $\varphi ,{\varphi }_{h}$ encourage temperature distribution.

## Future recommendation

Based on the findings of this investigation, it is suggested that further investigation be carried out to verify the results obtained through computer modeling and assess the viability of adopting CNT hybrid nanofluids and the Cattaneo–Christov heat flux model in industrial applications of solar-powered ships. These novel approaches are anticipated to make a significant contribution toward the development of energy systems that are both more efficient and more environmentally friendly. It has been proposed that future research has to concentrate on carrying out experimental experiments to validate the outcomes of computational models. This research could also investigate other kinds of nanofluids and models of heat flow to find out whether or not they have the ability to improve the heat transfer performance of solar-powered ships. In general, the results of this work give a solid foundation for future research in this area. They also demonstrate the promising potential of utilizing CNT hybrid nanofluids and the Cattaneo–Christov heat flow model to improve the operation of solar-powered ships.

## Data Availability

All data used in this manuscript have been presented within the manuscript. No data are hidden or restricted.
